# Artificial Neural Network-Assisted Facial Analysis for Planning of Orthognathic Surgery

**DOI:** 10.4317/jced.62088

**Published:** 2024-11-01

**Authors:** Lívia Mirelle Barbosa, Joana de Ângelis Alves Silva, José Ivson Soares da Silva, Tsang Ing Ren, Belmiro Cavalcanti do Egito Vasconcelos, José Rodrigues Laureano Filho

**Affiliations:** 1 Department of Oral and Maxillofacial Surgery, University of Pernambuco, Recife-PE, Brazil; 2 Information Technology Center at the Federal University of Pernambuco, Recife-PE, Brazil

## Abstract

**Background:**

Facial analysis for orthognathic surgery aims to identify facial features and determine how occlusion should be corrected to achieve facial balance. Several artificial neural networks have been developed to detect facial landmarks; however, no publications have reported the application of neural networks to measure facial proportions specifically for orthognathic surgery planning. Objectives: To develop a computer program that automates facial measurements through photograph capture, as well as to present the development stages of the program and the test results evaluating its effectiveness.

**Material and Methods:**

Researchers from the Postgraduate Program in Oral and Maxillofacial Surgery at the University of Pernambuco (UPE), in collaboration with researchers from the IT Center at the Federal University of Pernambuco (UFPE), developed a computer program to automate facial measurements through photographic capture.

**Results:**

The developed program successfully detected nine measurements: (M1) middle third of the face, (M2) lower third, (M3) intercanthal distance, (M4) alar base, (M5) upper lip, (M6) upper lip vermilion, (M7) lower lip, (M8) lower lip vermilion, and (M9) interlabial gap. Of these measurements, only two showed significant discrepancies (*p*<0.01) compared to the actual size in both versions of the program. These discrepancies referred to the middle third of the face and the lower lip vermilion.

**Conclusions:**

The developed program can be considered effective, as it automatically detected seven facial measurements relevant to orthognathic surgery. However, this line of research must be continued to create a larger database and train the network more robustly, increasing its ability to detect more facial landmarks and allowing the automated acquisition of additional measurements important for orthognathic surgery planning.

** Key words:**Dentofacial deformities, maxillofacial abnormalities, orthognathic surgery, artificial intelligence, software.

## Introduction

Facial analysis plays a crucial role in the identification of facial features and in the determination of how occlusion will be corrected to achieve facial equilibrium, and can serve as a tool for the organization, understanding and communication between orthodontist, oral & maxillofacial surgeon and patient (1,2).

The creation of a treatment plan in orthognathic surgery requires of the surgeon, in addition to surgical techniques, a knowledge of craniofacial anatomy and physiology, craniofacial deformities and their etiologies. However, it is known that machines surpass human beings’ capacity to store and organize information, consequently no surgeon would be able to outdo a computer with regard to memorizing data (3,4).

In this context, the field of artificial intelligence (AI) has been making huge advances, enabling computers not only to perform rudimentary cognitive functions, such as optical facial recognition, but also execute more complex cognitive tasks including the analysis and interpretation of a face identified by the computer (1,5). Therefore, AI appears to be a promising tool to support diagnosis and help in taking decisions, along with the surgeon, in the facial analysis stage.

The AI used in the recognition of images to enhance medical diagnoses is employed in various specialties. In oral and maxillofacial surgery it is used in the marking of points and the acquisition of cephalometric measurements, as well as the classification of tooth types in cone beam computed tomography images, diagnosis of oral cancer, facial phenotyping of genetic diseases, and evaluation of the effect of orthognathic treatment on facial attractiveness and apparent age (1,6,7).

Various AI neural networks have been developed to detect facial landmarks (8-18) however, as yet, no publications have been found describing the use of neural networks to take measurements of facial proportions with the aim of planning orthognathic surgery.

Given the lack of technological tools that enable the automation of facial measurements through the capture of photographic images, researchers from the

Department of Post Graduation in Oral & Maxillofacial Surgery at the University of Pernambuco (UPE), together with researchers from the IT center at the Federal University of Pernambuco (UFPE), have developed a computer program with this in mind. The aim of the present study is to present the program development stages and the results of tests to evaluate its effectiveness.

## Material and Methods

This study was conducted taking into consideration the ethical aspects advocated by Resolution CNS 466/12 of the Brazilian National Health Council in respect of ethics in human research, and was approved by the Research Ethics Committee at the University of Pernambuco under protocol no. 59375522.8.0000.5207. The participants were informed about the objectives of the study, as well as the risks, benefits and responsibilities of the researcher.

Development of neural networks to detect compatible facial landmarks for the measures studied in facial analysis for orthognathic surgery

This stage was developed by a doctorate student in the Post Graduation in Computer Science Program at the Information Technology Center of the Federal University of Pernambuco. It started out with studies and attempts to adapt different available facial recognition networks that generated landmarks on facial structures of interest for the measurements required for the planning of orthognathic surgery (8-18).

The facial landmark detection model was an adaptation of the Tweaked convolutional neural network (13) developed by researchers at the Information Sciences Institute at the University of Southern California. This neural network was used because it specializes in locating facial reference points in a variety of poses and countenances. Regressing Local Binary Feature methods were also used, developed by researchers at the University of Science and Technology in China (18).

Regressing Local Binary Feature methods possessed two components: a set of local binary resources and a principle of locality in order to understand these resources, as it independently permits the learning of a set of highly discriminating, local binary features for each facial reference point (18).

The local binary resources obtained were used to jointly learn a linear regression for the final output (18). These characteristics also allowed the network to perform the function of obtaining the necessary measurements for facial analysis prior to orthognathic surgery, as per the criteria described by Arnett (2) and Bergman, (1993).

The Tweaked convolutional network (13) was proposed for the localization of five facial landmarks, however, for the purposes of the present study, a minimum of 13 facial landmarks would be required. These facial landmarks are located in strategically anatomical positions that enable facial analysis measurements for orthognathic surgery. As a result, the need was perceived to create a database of photographs and facial measurements of a variety of individuals for the requisite network training.

Creation of database with measurements for facial analysis

Collections from 20 participants were carried out initially in order to calibrate the image capture parameters (device, distance, markers, poses). Following this methodological finetuning, a database was created involving 80 people aged between 18 and 60, of both sexes, with or without dentofacial deformities. Frontal and profile photographs were taken at a distance of 70 cm and a height of 120 cm. The Nikon D90 camera was positioned with the aid of a tripod and the participants were subjected to the capture of facial images and measurements in the sitting position.

**Figure 1:**
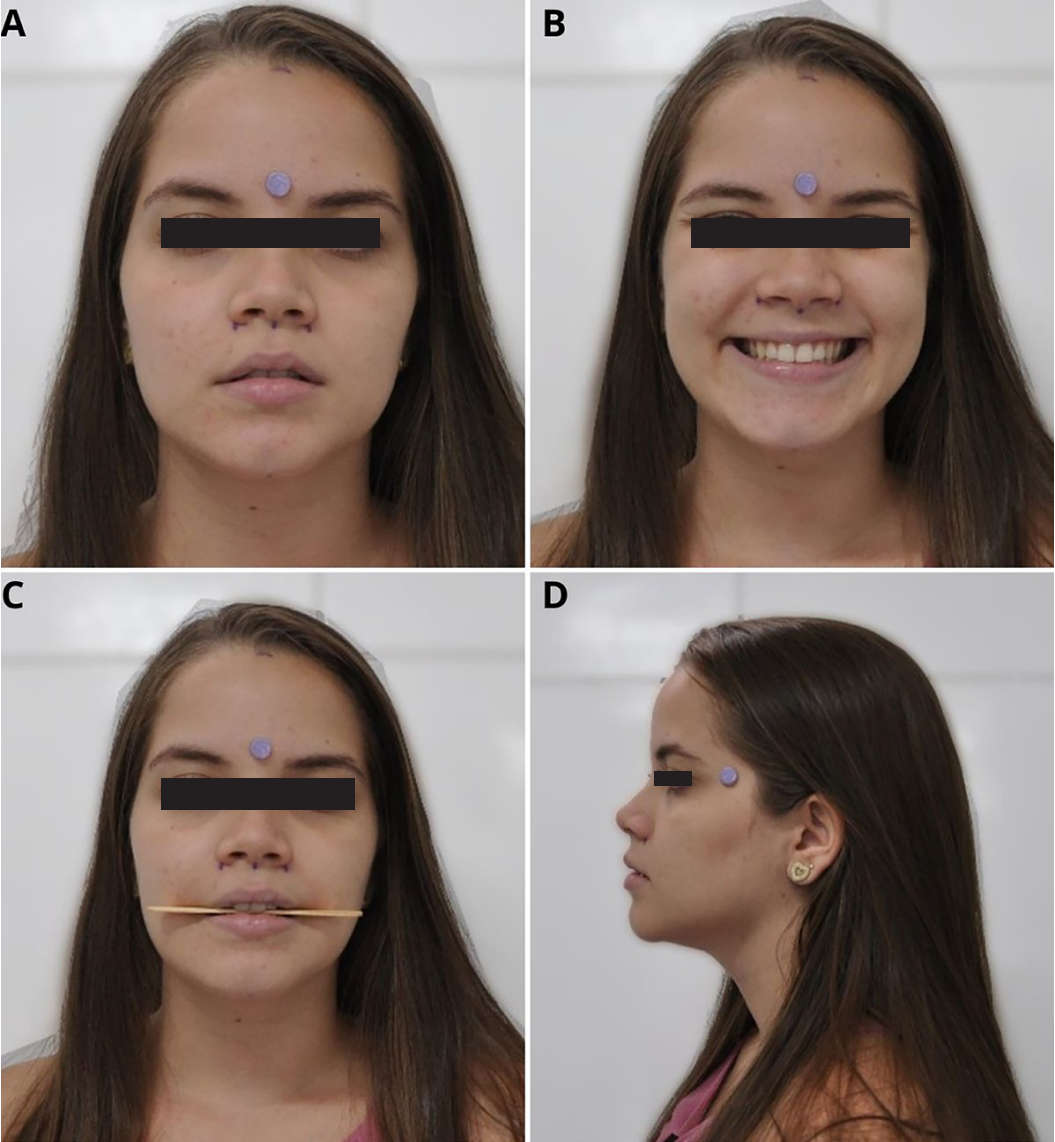
A: Frontal photograph, at rest, for creation of database for network training. B: Frontal photograph, while smiling, for creation of database for network training. C: Frontal photograph, biting wooden lollipop stick for creation of database for network training. D: Profile photograph, at rest, for creation of database for network training.

During the photographic session, a silicone adhesive pad (3M Impact Protection) measuring 1 cm in diameter was used, affixed to the upper third of the face in order to help with the calculations between actual size obtained using Absolute Mitutoyo digital calipers (150 mm x 0.01 mm - 500-196-30) and the size of the image. The frontal photographs included poses by the participant at rest (Fig. 1A), with a forced smile (Fig. 1B) and biting a wooden lollipop stick (Fig. 1C). The profile photographs were taken in a resting position (Fig. 1D).

For each participant, digital calipers were used to take measurements of the parameters cited by Arnett (2) and Bergman (1993), which are important to the planning of orthognathic surgery: 1) upper third, 2) middle third, 3) lower third, 4) intercanthal distance, 5) alar base, 6) upper lip, 7) upper lip vermillion, 8) lower lip, 9) lower lip vermillion, 10) interlabial gap, 11) exposure of upper central incisor, at rest, 12) exposure of upper central incisor with forced smile, 13) gingival exposure, at rest, 14) gingival exposure with forced smile, 15) length of upper central incisor, 16) chin-neck line, 17) distance between maxillary occlusal plane and lateral corner of left eye, 18) distance between the maxillary occlusal plane and lateral corner of right eye, 19) distance between facial midline and dental maxillary midline, 20) distance between facial midline and dental midline of the mandibular arch, 21) distance between facial midline and chin midline (pogonion).

The same parameters as cited above were measured on the photographic images using the GIMP software program (GNU Image Manipulation Program), permitting an association between actual size and image size. The association was made via the proportion of the distance between the facial landmarks in pixels and the pixel size of the circular, silicone pad, knowing that the measurement of this device was 10 mm.

Training to detect facial landmarks for the measurements studied in the facial analysis for orthognathic surgery

**Figure 2:**
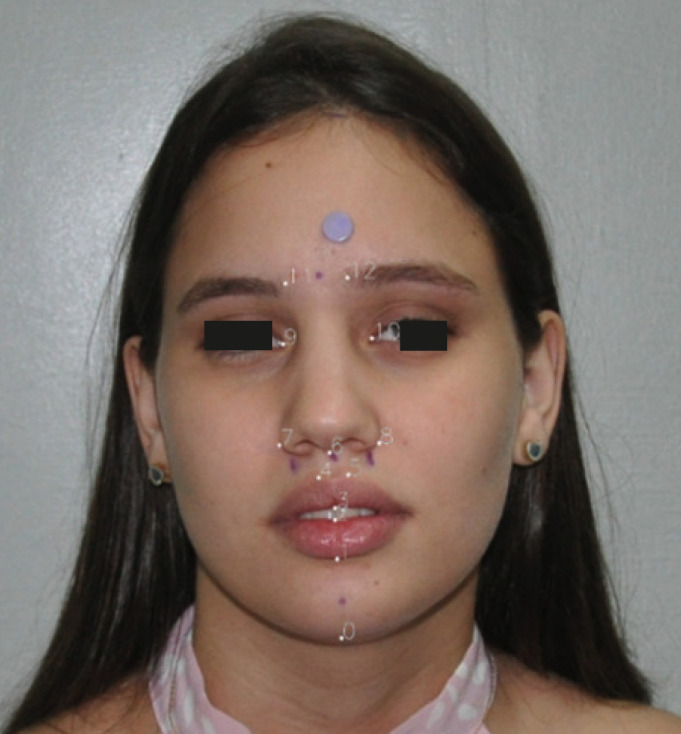
Numbering of facial landmarks in the marked positions. 0: Mentoniano, 1: UnderLipBotton, 2: UnderLipTop, 3: UpperLipBottom, 4: UpperLipTopRight, 5: UpperLipTopLeft, 6: Subnasal, 7: NoseRightAlarOutTip, 8: NoseLeftAlarOutTip, 9: EyeRightInner, 10: EyeLeftInner, 11: EyebrowRightInner e 12: EyebrowLeftInner.

Each image was cropped to 1024x1024 pixels with the face centralized. The data were subsequently incremented in order to increase the input variability, in which a horizontal inversion or a change in saturation were applied. The 13 points of interest were marked on a binarized mask that was used as the gold standard for training the network (Fig. 2).

**Figure 3:**
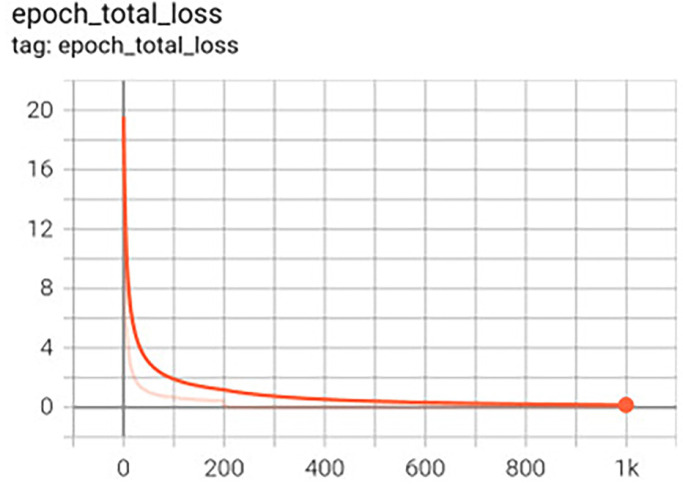
Graph of loss function during training.

For the network input, the image was resized to 40x40 pixels. The architecture is based on the Tweaked network (13), with a change in the number of facial landmarks from 5 to 13, however, the loss function and optimizer are the same, MSE (Mean Squared Error) and Adam19. The number of training epochs was evaluated for 200 and 1,000. This value refers to the number of times the images are presented to the training network, resulting in the creation of two network versions. The batch size was defined as 1 since, for larger batches, it was found that the normalization of loss function had no effect (Fig. 3).

-Clinical usage test of the application

The test group comprised 20 participants. This group comprised 10 men and 10 women, of whom 5 (2 men and 3 women) suffered from dentofacial deformities. The face measurements, obtained through conventional facial analysis, were compared to the measurements generated by two versions of the program: one version with 200 (version 1) and the other version with 1000 training epochs (version 2).

-Statistical method

To investigate agreement between the facial analysis methods, the statistical paired t-test was employed, which evaluates if the mean values of the two listed measurements are statistically different from one another.

The margin of error employed when deciding upon the statistical tests was 5%. The data were input to an Excel spreadsheet and the program used to acquire the statistical calculations was IBM SPSS, version 25.

## Results

**Figure 4:**
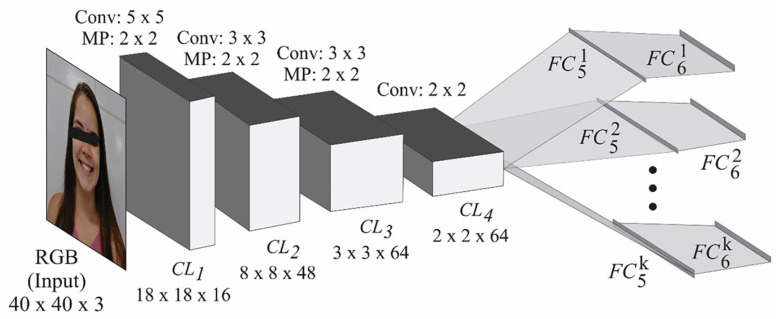
Neural network architecture, adaptation of the Tweaked convolutional network (13).

The neural network developed in this study comprises four convolutional layers, with one version having 200 training epochs and the other, 1000. In addition, for each facial point, there are more than two fully connected layers (Fig. 4). The result is 13 pairs indicating the position (x,y) of the facial landmarks on the image according to the Figure (Fig. 2).

The developed network, using the TensorFlow framework, can be exported to the TensorFlow Lite format and made available for mobile applications. The image input to the neural network should have a dimension of 40x40 pixels, with the face centralized.

Of the 21 facial measurements of interest for the planning of orthognathic surgery that were collected to build the database and for network training, the network managed to detect nine measurements: (M1) middle third, (M2) lower third, (M3) intercanthal distance, (M4) alar base, (M5) upper lip, (M6) upper lip vermillion, (M7) lower lip, (M8) lower lip vermillion, (M9) interlabial gap.

The face measurements generated by the two versions of the program were compared to the measurements obtained from the conventional facial analysis of 20 people.  Table 1 exhibits the mean and standard deviation of the differences between the facial measurement methods for the diagnosis and planning of orthognathic surgery: CM (conventional measurement), AM1 (automated measurement, version 1- with training in 200 epochs), AM2 (automated measurement, version 2- with training in 1000 epochs).

The analysis of  Table 1 enables us to infer that the actual measurement of the middle third of the face produced the biggest mean difference versus the measurements obtained through the two versions of the program.

According to the analysis in  Table 2, it is possible to see that, of the nine measurements detected by the program, just two were in disagreement (*p*<0.01) with the actual size in the two versions of the program. These measurements relate to the middle third of the face and the lower lip vermillion. The actual measurements of the lower third of the face, lower lip and alar base were only in agreement (*p*>0.05) with the automated measurement of version 2 of the program (i.e. with network training of 1000 epochs). The measurement of the upper lip vermillion was compatible (*p*>0.05) with the automated measurement in version 1 of the program (with network training of 200 epochs). The measurements of intercanthal distance, upper lip and interlabial gap were in agreement (*p*>0.05) with the two versions of the program.

## Discussion

The neural network developed in this study comprises four convolutional layers. These deep-learning techniques, based on Convolutional Neural Networks (CNN), were used in this study on account of their efficiency in the processing and analysis of digital images (21,22).

CNN is defined as a deep-learning algorithm capable of capturing an input image, attributing importance (weighting and bias that can be learned) to various aspects / objects in the image, distinguishing one from another. Compared to other classification algorithms, the preprocessing required in a CNN is much smaller. While in the primitive methods the filters are manually applied, with sufficient training the CNNs can learn these feature filters (23).

Despite the availability of various AI neural networks for the detection of facial landmarks (8-18), as yet, no publications have been located reporting on the use of neural networks to measure facial proportions with the aim of orthognathic surgical planning, nor in other areas of knowledge such as forensic techniques or orofacial harmonization. However, CNNs are described in the literature for the automation of cephalometric measurements (6) and for evaluation of the impact of orthognathic surgery on facial attractiveness and apparent age (1).

To carry out automated cephalometric analysis, Arik *et al*. (6)(2017) trained a CNN using a publicly available, cephalometric radiographic image dataset. In general, the results demonstrated high precision in the detection of anatomic points of reference and high levels of precision for the diagnosis.

The study by Patcas *et al*. (1) (2019) was a groundbreaker in the use of artificial intelligence to describe the impact of orthognathic surgery on facial attractiveness and apparent age. Pre- and post-treatment photographs of patients subjected to orthognathic surgery were collected for this retrospective, longitudinal study. For each image, facial attractiveness and apparent age were established with CNNs trained with images (1). According to the algorithms, the appearance of the majority of the patients improved with treatment (66.4%), resulting in them appearing to be almost a year younger, and also with an improvement in attractiveness of 74.7%, principally after mandibular surgery (1).

The study by Yan *et al*. (24) (2018) developed a method based on end-to-end, deep-learning training that carries out mandibular segmentation in an automated fashion. Unlike CNN, the team worked with a symmetric convolutional neural network (SCNN). This enabled the computation of convolution and deconvolution to be symmetrical in order to achieve good segmentation performance. Moreover, it reduced the level of human effort and was able to achieve competitive performance. The experimental results showed that the proposed SCNN is superior to various popular databases in terms of the data similarity coefficient (24).

Even though CNNs have some applicability in different topics of oral and maxillofacial surgery, the lack of CNNs focusing on facial analysis prior to orthognathic surgery makes it impossible to compare the results of the present study with those of other studies. Nevertheless, this fact underlines the innovative and pioneering nature of this study.

The initial goal when developing this network was to achieve automation of 21 facial measurements of importance for the diagnosis and planning of orthognathic surgery. However, the developed network detected nine measurements. This fact attests to one of the main difficulties encountered in network development, namely finding a network that can detect facial landmarks in anatomical areas that favor the measurement of parameters important for orthognathic surgery.

Despite the availability of various AI neural networks for detecting facial landmarks (8-18), the anatomical localization of the points detected by these studied networks did not permit precise detection of the measurements of interest for the planning of orthognathic surgery. To circumvent this obstacle, it was necessary to adapt the Tweaked network architecture (13) and train the new network by positioning the facial landmarks in anatomical areas that made it possible for the program to detect the nine measures.

The program that was developed may be considered to have performed well, given that it succeeded in automatically detecting seven facial measurements of interest to orthognathic surgery. However, this line of study must be continued so that a larger database can be created and the network can be trained in a more robust way, increasing its ability to detect more facial landmarks, permitting the automated acquisition of further measurements of importance to orthognathic surgery. Once these difficulties have been resolved, this line of study can go ahead and export the network to the TensorFlow Lite format made available to mobile applications.

The future of this research will involve the development of an application that will facilitate the clinical work of the Oral & Maxillofacial Surgeon in the facial analysis stage so that the application can capture images of the patient and automatically generate measurements of interest to orthognathic surgery.

## Figures and Tables

**Table 1:** Mean and standard deviation of differences between facial measurement methods for the diagnosis and planning in orthognathic surgery: CM and AM1, CM and AM2 for measurements M1 to M9.

Measurement	CM – AM1	CM – AM2
	Mean ± SD	Mean ± SD
M1. middle third (n = 20)	6.14 ± 5.32	6.38 ± 6.08
M2. lower third (n = 20)	3.47 ± 6.55	1.58 ± 7.07
M3. Intercanthal distance (n = 20)	0.01 ± 2.90	-0.81 ± 3.01
M4. Alar Base (n = 20)	-1.71 ± 3.41	-0.99 ± 4.18
M5. Upper lip (n = 20)	1.32 ± 3.18	1.01 ± 3.51
M6. Upper lip vermillion (n = 20)	0.33 ± 1.61	1.15 ± 2.04
M7. Lower lip (n= 20)	2.82 ± 5.56	1.66 ± 4.98
M8. Lower lip vermillion (n = 20)	2.44 ± 1.68	1.74 ± 1.62
M9. Interlabial gap (n = 20)	-0.13 ± 1.15	-0.61 ± 1.43

CM: conventional measurement AM1: automated measurement version 1- with network training in 200 epochs AM2: automated measurement version 2- with network training in 1,000 epochs

**Table 2:** Mean and standard deviation of measurements M1 to M9 according to the facial measurement methods for diagnosis and planning in orthognathic surgery.

Measurement	CM	AM1	AM2	P Value^(1)^	P Value^(2)^
	Mean ± SD	Mean ± SD	Mean ± SD		
M1. Middle third of face (n = 20)	62.47 ± 5.05	56.33 ± 2.50	56.09 ± 3.06	p(1) < 0.001*	p(2) < 0.001*
M2. Lower third of face (n = 20)	67.42 ± 6.22	63.95 ± 3.94	65.84 ± 3.62	p(1) = 0.029*	p(2) = 0.330
M3. Intercanthal distance (n = 20)	32.02 ± 2.41	32.01 ± 2.30	32.83 ± 2.40	p(1) = 0.983	p(2) = 0.244
M4. Alar Base (n = 20)	33.36 ± 3.54	35.08 ± 2.59	34.35 ± 3.60	p(1) = 0.037*	p(2) = 0.304
M5. Upper lip (n = 20)	20.55 ± 3.08	19.23 ± 1.30	19.54 ± 1.33	p(1) = 0.079	p(2) = 0.216
M6. Upper lip vermillion (n = 20)	9.27 ± 1.35	8.94 ± 1.01	8.12 ± 1.51	p(1) = 0.366	p(2) = 0.021*
M7. Lower lip (n= 20)	45.21 ± 4.79	42.39 ± 3.35	43.55 ± 2.93	p(1) = 0.035*	p(2) = 0.152
M8. Lower lip vermillion (n = 20)	11.96 ± 1.88	9.51 ± 1.01	10.22 ± 1.60	p(1) < 0.001*	p(2) < 0.001*
M9. Interlabial gap (n = 20)	2.20 ± 0.93	2.34 ± 0.59	2.81 ± 0.98	p(1) = 0.610	p(2) = 0.072

(*) Significant difference at level of 5.0% (1) Student t-test for comparison of CM and AM1 (2) Paired student t-test for comparison of CM and AM2 CM: conventional measurement AM1: automated measurement version 1- with network training in 200 epochs AM2: automated measurement version 2- with network training in 1,000 epochs

## Data Availability

The datasets used and/or analyzed during the current study are available from the corresponding author.
